# Agency: What Does It Mean to Be a Human Being?

**DOI:** 10.3389/fpsyg.2021.693077

**Published:** 2021-09-20

**Authors:** Richard N. Williams, Edwin E. Gantt, Lane Fischer

**Affiliations:** ^1^Department of Counseling Psychology and Special Education, Brigham Young University, Provo, UT, United States; ^2^Department of Psychology, Brigham Young University, Provo, UT, United States

**Keywords:** agency, free will (freedom), Hayek F. A., modernism, postmodernism, positivism

## Abstract

This paper will look at the results of what has been termed “the crisis of modernism” and the related rise of postmodern perspectives in the 19th and 20th centuries. It concentrates on what is arguably the chief casualty of this crisis – human agency – and the social science that has developed out of the crisis. We argue that modern and postmodern social science ultimately obviate human agency in the understanding of what it means to be a human being. Attention is given to the contemporary intellectual world and the way in which it has been deeply informed by neo-Hegelian and other postmodern scholarly trends, particularly in accounting for how agency has come to play little role in social science understanding of human action. The paper also offers an alternative conception of human agency to the commonly endorsed libertarian model of free choice. Finally, the paper argues that this view of agency preserves meaning and purpose in human action and counters the pervasive social science worldview that sacrifices agency and meaning to powerful invisible abstractions.

## Introduction

It has frequently been argued (see, e.g., Gay, 1969; Outram, 1995; Beiser, 1996; Gottlieb, 2016; Pinker, 2018; Williams and Gantt, 2018) that Enlightenment thought, as it matured from the early 16th century and on through the late 19th century, exalted the rational mind as the source of all knowledge worthy to be deemed real knowledge. The finished form of this creative rational process was thought to be found in formal logic and scientific discourse that could be shown to embody or pass the test of careful logical analysis in its structure, claims, and conclusions. This logic test, in more recent centuries, has also assumed the form of empirical demonstration, validation, or falsification. In our contemporary intellectual climate, it seems that such empirical demonstrations take their most impressive form, in the activities of the natural sciences and the technologies they produce (Wooton, 2015).

Rational science, as it developed, offered the promise of control over nature in the service of humankind on such a scale that a great host of human needs seemed finally on the brink of being met. As more human needs were met, the success of such control and technology gave impetus (and perhaps even lent legitimacy) to a sometimes subtle and unnoticed turning of attention away from human needs that had hitherto been largely physical and economic and toward human wants, many of which became mental, psychological, and emotional, or what we might term “mere desires.” With tangible needs largely fulfilled – at least in principle – it seemed conceivable and legitimate to turn attention to the fulfillment of mere desires. In a sense, this historical success of Enlightenment thinking has in every subsequent era contributed to the appearance that the earliest promises of the Enlightenment, articulated perhaps most famously by Renaissance philosopher Giovanni Pico della Mirandola (1,463–1,494), might actually be fulfilled. In his work, *De Hominis Dignitate* (On the Dignity of Man), Pico della Mirandola attributes to God the following description of our human nature:

We have set thee at the world’s center that thou mayest from thence more easily observe whatever is in the world. We have made thee neither of heaven nor of earth, neither mortal nor immortal, so that with freedom of choice and with honor, as though the maker and molder of thyself, thou mayest fashion thyself in whatever shape thou shalt prefer (cited in Cassirer et al., 1948, pp. 223–225).

In the following paragraph in this essay, Pico della Mirandola expresses one other aspect of our nature for which, he suggests, we all ought to be properly grateful: “O supreme generosity of God the Father, O highest and most marvelous felicity of man! To him it is granted to have whatever he chooses, to be whatever he wills” (cited in Cassirer et al., 1948, pp. 223–225). In this passage, written at the earliest phase of the Enlightenment, we can see already uncovered the end to which Enlightenment thought aimed to carry humankind, and to which, to a great extent we still continue to aspire (see, e.g., Bellah et al., 1985; Wilkens and Sanford, 2009; Tallis, 2020).

The progress of the Enlightenment project, as begun in earnest in the 16th century and developed throughout the 18th and into the 19th century, surely raised the collective expectations of the Western world. The power of the rational mind to discover and to create in virtually every aspect of nature and every field of endeavor seemed limitless – in theory, at least, if not always quite yet in practice. Enlightenment rationality was applied to the questions of epistemology, to the subjects of natural philosophy, and to the dilemmas of moral philosophy. Indeed, to many, it seemed that every human question and problem would 1day fall to the rational powers of the human mind (Gay, 1969). The invention of increasingly sophisticated methods of study, of observation and measurement, drew out of natural philosophy, a set of practices, both methodological and explanatory, that coalesced into the natural sciences of the 19th and 20th centuries and subsequently validated themselves in the form of the technologies of the 20th and 21st centuries (McClellan, 2015). Sophisticated processes of formal logic increasingly held out the possibility of rational certainty about the world and even about ourselves. Indeed, it was not clear that there were any predetermined limits to our capacity to know with absolute confidence virtually anything to which we might turn our collective minds (Pinker, 2018). Even moral philosophy, reaching into theology, underwent transformations as the tools of rational analysis were applied to moral principles and religious doctrines (Olson, 2013). The effect in this sphere was refinement of religious argument and new forms of apologetics, on the one hand, and more sophisticated forms of scientism, naturalism, skepticism, and atheism, on the other (Olson, 2008).

By the early to middle 20th century, however, some very insightful and sophisticated voices began to question, with greater seriousness and sophistication than in earlier centuries, the hegemony and even the relevance of the rationalist project, and its ability to really make sense of all things – most especially, its ability to make sense of our humanity (Hayes, 2009). Europe had been the seedbed of the Enlightenment, and science, technology, political philosophy, art, and moral theory all bore the marks of the Enlightenment and its European cultural context (Gay, 1969). Thus, Europe was not only the seedbed of Enlightenment thinking, but it was also the bearer to the world of the intellectual, political, and technological fruits of that thinking. Unfortunately, despite the seemingly utopic promises of Enlightenment science and philosophy, the late 19th through the middle 20th centuries saw unprecedented turmoil and strife, including world wars that produced greater destruction and human suffering than anyone, from the perspective of the high point of rationalism in the 18th and early 19th centuries, could ever have predicted. This led some thinkers, beginning in the interwar period of the 20th century, to begin questioning the aims and legitimacy of the modernist project – arguing quite thoughtfully and persuasively that, for all of its success in furthering our understanding of the natural world, modern science and philosophy had failed to provide answers to the most pressing moral and existential questions human beings face, especially those regarding the very the nature of our humanity itself. In short, Enlightenment modernism had failed to safeguard the inherent meaningfulness of the human world. Thus, a rich and penetrating philosophical literature grew up centered on an analysis of the widely recognized “crisis” of the modern world (see Sharpe et al., 2017) and the resultant “malaise of modernity” that the issues at the core of the crisis subsequently engendered (Taylor, 1991; Gantt and Williams, 2018).

## The Intellectual Landscape Following the Crisis

The crisis we are referring to here was a crisis of meaning, the inability to be sure about, or to find a stable ground for the human meaning-making and understanding that provides the necessary structure for pursuing science, philosophy, moral theory, art and culture, theology, and for creating and sustaining those social institutions founded on the rationalist tradition that had dominated Western thought for the previous three centuries. While the concept of “crisis” was primarily developed through the writings of the German philosopher Edmund Husserl (1859–1938) and likeminded others (see, Hewitson and D’Auria, 2012) during the interwar period in Europe, the origins of this crisis in meaning, and the larger perception of the possible dangers of modernist rationalism, began to emerge earlier in the 19th century, in the writings of Søren Kierkegaard (see, e.g., analyses by Pattison, 2002 and Stewart, 2015), Fyodor Dostoevsky (e.g., *The Idiot*), and Nietzsche (2002), but was perhaps most visible in the rise of such intellectual movements as Marxism, Darwinism, and, slightly later, Freudianism. Indeed, possibly the most sweeping and ambitious form of the Enlightenment project of modernist rationalism is the work of G. W. F. Hegel (1770–1831) in the early 19th century. In a sense, Hegel moved Enlightenment rationality forward from the original epistemic hegemony of the individual rational mind to the postulation of Mind (or Spirit or *Geist*) itself as the ultimate explanation of all that is – including human beings and our individual minds (Dale, 2014). Hegelianism was, in an important sense, the implicative endpoint of modernism as Enlightenment rationalism. This is so, in large part, because Hegelianism brought together in one grand system two of the fundamental questions of rationalism (and really, of the Western tradition itself): epistemology and metaphysics. The implications of this grand system for the third pillar of Western intellectual life (i.e., ethics and moral theory) have been significant and far-reaching.

The 20th-century philosopher, historian, and economist, Friedrich Hayek (1899–1992), provides what we believe is an important account of the intellectual history of the post-Enlightenment or “postmodern” period. In his book, *The Counter-Revolution of Science: Studies in the Abuse of Reason*, Hayek (1979) proposes that, although it is a great irony, the dominant thought of the late 20th century, and thus of the early 21st century, has been shaped by a conceptual alliance – perhaps a coalescence – of the rationalism of Hegel (or, the Hegelians) and the positivist thought of Auguste Comte (1798–1857) and his followers. The common element that allowed two such seemingly disparate intellectual traditions to come together as a genuine intellectual force in the 20th century and to continue up to the present time is, we argue, the willingness of both traditions to postulate the existence of certain powerful unembodied and timeless entities, constructs, or abstractions, capable of exerting substantive causal power over – and, thereby, governing and determining – events and entities in the real, material, temporal world that human beings inhabit. The power of these causal abstractions includes not only a controlling force (once underway), but also a creative force – or, expressed in terms more acceptable to proponents of this explanatory tack, a “constitutive power.” This is to say that, one way or another, the abstract, universal reality, or realities (e.g., constructs) – which are the products of both Hegelian and positivist thinking – make both things and events, including behaviors, thoughts, and feelings, what they are and how they are. All things come from – that is, all things are constitutive of – a higher, more abstract, more fundamental reality. It is worth noting here that the first and chief casualty of this ontologic–epistemic system is human agency itself (Dyde, 1894; Hayek, 1979).

To many students of the history of ideas, the possibility of the extreme rationalism of Hegel and his successors coming together coherently with the positivism of Comte and his successors may seem unlikely, if not impossible. Hayek clearly understood the strangeness of the proposition that these two schools of thought should come together and dominate 20th (and 21st) century’s thought and explanation. Nevertheless, he makes a compelling case that such has indeed taken place. If looked at from a slightly different angle, however, this proposition is not terribly surprising. After all, both positivism and German idealism are what we might call “theories of everything” (Barrow, 1991). In substantive ways, both of these traditions represent the ultimate project of the Enlightenment.

Taking one’s route through philosophy, one ends up in the radical idealism descended from Hegel, wherein all is explained by the clash (or evolution) of ideas, which move both themselves and the events that constitute human reality. Truth and knowledge may reside in the individual mind, but they exist supra-personally at the highest and broadest possible level of reality. Hayek used the term “supermind” to refer to this elevation of the nature and function of the modern enlightenment conception of mind, and its creative power and receptivity to knowledge, to a cosmic level that affords – to those who can grasp the system and its evolution – understanding of the whole of the “system” of ideas at work and moving itself toward its own ends. Hayek (1979), it should be noted also uses the term “supermind” to refer to those individuals (the Intelligentsia) who have been able to grasp the reality of the workings of the grand system and who thus have the power to work with it and further its inevitable end. According to this line of thought, which has great currency in these early decades of the 21st century, abstract realities are the real source and cause of things. Thus, events and reality are known best and most fully as, or by means of, constructs and structures that exist, are on the move, and can be understood only at that abstract level. The individual mind knows by contacting and apprehending what is fundamentally an abstract reality.

The other grand achievement of Enlightenment thought is, of course, modern science. And, thus, taking one’s route to contemporary social theories and models of humanity through science, one ends up in some form of positivism – or, at least, positivists would have us understand positivism, or positivistic science, as the end point of just that epistemic certainty that has been held to be the standard of truth and knowledge in Modernist thought (Ayer, 1959). Positivism allows into the discourse and practice of science a number of powerful abstractions, which serve as an endpoint of science and explanation – even when they are not directly detectable or demonstrable. Such abstractions include constructs, laws, processes, principles, and structures. In positivist thought the things and events we encounter in the empirical world are best and most fully explained and understood in terms of such abstractions – by laying such abstractions “over top of” the empirical events of the world in order to produce coherent (typically causal) accounts of empirical events. The “goodness of fit” of the events under the umbrella provided by the abstract constructs, structures, and systems is taken as evidence for the reality and existence of the constructs, laws, structures, and systems themselves. Even when, as is often the case in contemporary social science, one is reluctant to use the language of “laws” to account for the human phenomena under study, the “regularities” that positivistic scientific investigations uncover are often taken to be “real” in some nontrivial sense, as the facts of the universe, so to speak – certainly more real than the specific empirical events or relationships that we observe, and that the abstract regularities are invoked to explain. Such regularities are taken to be lawful in nature, that is, governed by something very abstract and outside the world of material reality and, thus, certainly “law-like” even if we wish to avoid the language of “laws.”

The important thing to note here about this cohesion of German idealism and positivism is that they both seek – and make readily available a language of – causal regularity (or lawfulness). In the most basic terms, (both Hegelian idealism and positivism) see powerful unembodied abstractions as real or names of things that are real enough to cause other events to happen (or to be responsible for their happening) in the material human world – as well as in the immaterial human world of thought, emotion, and desire. A central question – certainly what should be *the* question at the base of much activity in the contemporary social sciences – is just how these abstract realities are to be known, uncovered, or adequately captured and controlled. The subsequent, and perhaps the ultimate, next step for both idealists and positivists, then, is determining how these powerful abstractions, once known, can be controlled and deployed in the service of resolving problems and, thereby, creating or manufacturing a better human reality than the one that we have now – built around individual human rationality and agency. Because both German idealism and positivism presuppose that human beings are mostly living in ignorance of the fuller reality of the causal abstractions at work in the world and in their lives, both traditions require a specialized degree of academic sophistication and even intellectual transcendence in order to apprehend with clarity the powerful abstractions they “discover” or presuppose. Obviously, “getting at” or comprehending these powerful abstractions is not easy; it requires deep and searching investigation – something clearly beyond the capability of normal, garden-variety human beings. Thus, the necessary level of insight and understanding will be available only to an educated elite who have cultivated the capacities to reach the level of understanding in which the powerful abstractions can be known and ultimately controlled – in the service of the betterment of humankind, of course.

## Hayek and the Supermind

What is required for this type and level of understanding requires invoking, in one way or another, what Hayek (1979) refers to as the “supermind” (p. 159). For Hayek, the supermind is straightforwardly derived from Hegelian thought. Any student of the history of ideas will understand that there is a sense in which Hegel moved explanation and understanding away from, or “outside of,” any and all particular, individual, rational minds and sought it in some sort of universal mind, *Geist* itself. The world must, thus, be finally understood as a product (in some sense) of a universal (or universalized) reason (i.e., thought). This process of ultimate reason that produces knowledge, truth, and understanding is described as the process by which “God comes to full consciousness of himself (becomes, i.e., Absolute Spirit)” (McCormack, 2000; p. 102). It is in this light that we commonly understand Hegel’s meta-historical process of *thesis* → *antithesis* → *synthesis*.

What Hayek is suggesting is that this process is presumed – by both idealists and positivists – only to be discernible at the level of a “supermind” and in terms transparent to, the supermind itself. Thus, true understanding of ourselves and our world requires a supermind, actualized in and by an educated elite whose consciousness of “what is really going on” has been sufficiently raised and expanded to permit them to grasp the higher order, systemic processes, and realities that really move events and govern human affairs, and which are opaque to almost all of the rest of us. Sustained deep thought, intellectual acumen, and unyielding commitment to rational methods and the findings such methods produce are required to attain contact with the underlying, abstract reality that constitutes the world, the grasping of which represents achievement of true understanding.^1^ Presuming all knowledge to be consilient, this will hold true throughout the human sciences, including psychology, sociology, political science, history, and across all social institutions, including family, government, religion, and so forth. Thus, it is believed that there is a great need for scholars and technicians who have the intelligence to master the intricacies and subtleties of the underlying (or overarching and ultimate) reality of the world and how it is enacted within and how it governs human affairs. These individuals, capable of rationally ordering, governing, and explaining the world in light of their superior knowledge and insight constitute the supermind, while almost all other (non-enlightened) human beings, because they are oblivious to what is really going on around them and within them – and why it is *really* going on – are relegated to being subject to the planning, oversight, and governance of the supermind (i.e., the rational and scientific intelligentsia).

It is at the level of the supermind (speaking for the enlightened body of scholars), and our confidence in its abilities, that the seeming improbable melding of German idealism (as the capstone of Enlightenment thinking) and Comtean positivism takes place. On a global level, one sees some intellectual kinship between the cosmic process of *thesis* → *antithesis* → *synthesis* as the legacy of Hegel and the Three-Stage Theory of cultural progress proposed by Comte (1988). Despite clear differences in theoretical details, both systems necessarily entail the same functional implications. As one of Hayek’s biographers, Ebenstein (2001) notes:

Hayek’s view of Hegel was similar to his perspective of Comte. He observed the paradox of joining Hegel and Comte, for the former is usually considered to be an idealist, and the later a (material) positivist. There was, however, little functional difference. For both, history moved in stages above and beyond the individual and removed from his will. What Hayek terms “historicism” is the mistaken belief that there are laws of history as there are laws of nature. Almost by definition, historicism denies moral standards, for it denies free will. Comte’s and Hegel’s determinism followed from their “peculiarly unhistorical approach to history,” perceiving determinism where it does not exist (p. 109).

In reducing human beings and their history to mere manifestations of the impersonal operations of powerful invisible abstractions, discernible only by those who have been properly trained to perceive such things, Hegel, Comte, and their intellectual descendants systematically obviate a central feature of human experience and daily life (i.e., agency).

## Agency: the First Casualty of Abstractions

Thus, the first casualty in the triumph of modern contemporary social theory is, indeed, any meaningful concept of human agency. At best, one is left with an empty Hobbesian concept of agency as being able to do what one wants (particularly in order to avoid unpleasant death or pain), despite the fact that our wants, which are the defining manifestation of our agency, are not of our own construction (Gantt and Williams, 2019). Additionally, one also senses strongly that similar sorts of training and education are required within both neo-Hegelian and positivist perspectives in order to fully “see” and “understand” the march of progress each school of thought describes. It is easy to see how and why post-Comtean positivists (including especially Comte’s students) saw Hegelians as allies and a certain kind of Hegelianism as part of the same intellectual project (Hayek, 1979).

At the same time, Comtean positivism has long been credited with introducing into science (including empirical science) the concept of *constructs*, hypothetical entities that enter the empirical process of science most directly at the explanatory level. The role of constructs is in suggesting, in terms that “make sense,” what forces might be at work in a particular (experimental) setting and then in suggesting how we can account for the results observed in a particular scientific study. *Constructs* are also manifest in terms of the operation of the definition and operation of *variables* in the events occurring during the experiment. Often, *constructs* are endowed with real causal efficacy at the level of models and theoretical explanations. Neither variables nor constructs can be detected by the sensory processes at work in the lived human world where most scientific experiments, including particularly social scientific experiments, are carried out. They are invisible to us as common human beings.

For example, one never sees a “stimulus”; one sees only light, objects, shapes, etc. The status of these real-world things as merely particular manifestations or instances of some underlying stimulus or combination of stimuli is a matter of conceptual inference. In other words, that such things are not actually taken to be what they appear to be, but are rather presumed to be examples of “stimuli” is a theoretical claim, not an empirical, scientific one (Williams, 1990). At the same time, we never observe or detect “responses.” Rather, we observe only events; that such event are taken to be “responses” is equally a theoretical claim. In every common use of these constructs in social science, the abstract “stimulus” has been endowed with a real causal power to enable it to bring about a discernible real-world event. This whole process is so common and feels so natural in the social sciences that we seldom stop to think that in this seemingly innocent process, we literally have created an unembodied abstraction and endowed it with causal efficacy in the real world. Furthermore, it is commonly assumed that anyone not sufficiently educated to see the world in terms of “stimuli” and “responses” will be in some important way ignorant and unable to understand what is really occurring.

An excellent example of precisely this sort of thinking can be seen in Milgram’s (1992) description of the nature of social psychological inquiry. “The creative claim of social psychology,” he writes, “lies in its capacity to reconstruct varied types of social experience in an experimental format, to clarify and make visible the operation of obscure social forces so that they may be explored in terms of the language of cause and effect” (p. xix). Similarly, he notes:

The common view is that social psychologists derive their experiments from life, and there is an important measure of truth in this. But it’s also true that events, such as the Genovese case, are the inevitable unfolding of forces that experimental analysis will frequently pinpoint first. Underlying the silly incident in the restaurant was an important principle of social behavior; by focusing on that latent principle, and extending it through to a concrete dramatized experiment, one could foresee certain inevitable results of such a principle (p. xxxi).

The genius of social psychology according to Milgram, then, is that it can “make visible the operation of obscure social forces” that undergird and govern human action and experience, and which are in themselves, unless discerned and exposed by one who has been properly trained, invisible, and inscrutable to the ordinary mind.

Although this example drawn from the social psychology of individual behavior is quite simple, the process involved and its conceptual effects are exactly the same in more complex and higher order situations when large-scale constructs such as “sexism” or “identity” are declared to have the real causal power to produce real tangible effects – the agency of human beings and their determination to do otherwise notwithstanding. If the causal abstraction is, “there” the resultant effect will occur. In this way, causal abstractions ultimately obviate and thus destroy human agency. However, we are assured by qualified and trained scientists who have studied such things that variables and constructs are indeed real and are the basis of the causal world we inhabit (see Addis et al., 2019). Even when not explicitly stated, examples of the presumption of the real existence of these abstractions and their causal efficacy are abundantly evident in the theoretical and methodological language of the social sciences. For example, it is present in the nearly ubiquitous obsession psychologists have with discovering the “main effects” and “interaction effects” that can presumably be revealed through careful analyses of variance and other forms of statistical examination, as well as in the extensive attempt to map the magnitude of between-person differences as indicators of the causal strength of these interactions (for a more detailed account of this issue and its many problems, see Lamiell, 2019). The interactions of principle interest are the interactions assumed to occur between various abstractions and the causal effects such interactions produce are particular human behaviors. In the social scientific literature, these powerful abstractions are known variously as laws, processes, principles, forces that work in and on us as “motivations,” “drives,” “needs,” “reinforcements,” “impulses,” “attractions,” “orientations,” “attitudes,” “stereotypes,” “traits,” “characteristics,” “schemata,” “scripts,” “structures,” “systems,” and so forth. In simplest terms then, German idealism and Comtean positivism have come together in the postmodern era due in large part to their shared metaphysical commitment to explaining the phenomena of human being in terms of powerful abstract entities possessing causal efficacy in human affairs at every level from the individual and internal to the external and global – or even cosmic. As both of these late-Enlightenment traditions came into contact with and were appropriated by postmodern thinkers, one of the principle results was the broadly defined “structuralist” movement – an intellectual movement that seemed to hold great promise for the humanities and the social sciences in the mid-1970 to late 1970 and which profoundly influenced cognitive psychologies, interpretations of Freudian psychologies, linguistic theories, and sociologies (see, e.g., Williams, 1978). Perhaps the most notable example of this movement was the resurgence of Marxist explanations for less economic or political and more local social and interpersonal phenomena. It is interesting to note that, in the opinion of some, the most lasting contribution of Marx’s philosophy was not in the area of political or even economic theory, but rather in epistemology. Marxism is perhaps the clearest and most accessible manifestation of the confluence of rationalism/idealism and positivism – only those trained to see the world in neo-Hegelian, neo-Marxist structural and systemic terms can really understand the phenomena of the human world. Thus, within this contemporary movement, the epistemological task is really the provision of training in recognizing abstract and causally efficacious structures and systems.

Granted, many scholars have suggested that we are now in a “post-structuralist” world, that we have gone beyond structuralist accounts and models. We question, however, whether this claim can be true since the essence of the structuralist account is the reality, causal efficacy, and enlightening power of unseen abstractions – be they structures, systems, processes, or whatever else. It is also unclear just what intellectual movements have captured the field previously held by structuralism when so many structural explanations (such as Marxism, some versions of feminism, Darwinism, and any number of other “isms”) still enjoy such contemporary currency, both at the theoretical and meta-theoretical level and at the experimental level of variables, principles, and models of all sorts.

Finally, while many structuralists have traditionally eschewed the language of efficient or Newtonian causality, it is difficult to find any evidence that contemporary explanations of human phenomena in the social sciences are not causal in any real or substantive sense. In fact, we see no evidence that the social sciences have ceased to offer explanations obtained by virtue of their presumed ability to achieve contact with some external rational order or reality, either by immersion and sensitivity training or consciousness raising, on the one hand, or by rigorous scientific observation, on the other. Here, we propose, lies the seedbed of scientism in its many, varied contemporary manifestations. Scientism embodies a thorough-going commitment to a metaphysic of powerful, unseen, abstract causes – usually in the guise of materialist naturalism – as necessary to any and all legitimately scientific examinations or explanations (Sorell, 1991). Explanatory tacks invoking abstractions with causal efficacy are not really different in any important sense – particularly if one is concerned with the possibility and preservation of genuine human agency.

## Human Agency in the Enlightenment and Beyond

As we have argued, one of the principle casualties of causal explanations grounded in either material substances or abstract, invisible causes is the possibility of any sort of meaningful human agency (see, e.g., Hayek, 1978). The most basic conception of human agency is “meaningful, purposive self-direction” (see, e.g., Williams, 1992, 2005, 2017; Slife and Fisher, 2000; Martin et al., 2003; Frie, 2008; Yanchar, 2011, 2018; Gantt and Williams, 2014). Human agency is, however, quite helpless, or at least hapless, in the face of powerful invisible, causally determinative forces. The more such causally efficacious constructs or causes there are, and the more arcane they are – insofar as they are available only to the intelligentsia or supermind – the less agency and freedom there is available to the mass of humanity. Ironically, it is not clear just how the intelligentsia themselves might acquire for themselves any genuine agency even as enlightened supermind, but it seems to be an article of faith that when properly enlightened, one is able somehow to harness the power of the causal nexus that is the human world and purposefully further its inevitabilities to one’s own purposes (a possibility anticipated by the ancient Epicureans). Perhaps this is as close to human agency or autonomy as one can hope to get in our neo-Hegelian modern/postmodern world. It is worth asking, however, whether there is a path to genuine human agency within the dominant metaphysical and epistemological regime, given its allegiance to, or even, insistence on, a “metaphysics of things” (Williams, 1990) – abstract things possessing causal efficacy. A brief historical review is, perhaps, the best way into this important question.

It is helpful to first point out that our contemporary conception of human agency – conceived of as an autonomous free will – is a product of Enlightenment thinking and, therefore, a relatively new concept (see, Taylor, 1989). We will say more about this below. In the classical Greek tradition, what we would recognize as human agency took the form of the active powers of self-direction (Frede, 2011). While this is not unrelated to our modern conception of agency, there are important differences with the ancient view, but also shades of common understanding, not the least of which is a fairly close tie between the powers of self-direction and moral considerations – that is, the pursuit of happiness in the form of the Good and furthering the Good in ways available to us as the kinds of beings we fundamentally are. We see here, of course, the roots of what we recognize today as virtue ethics. As early as the Pre-socratics, the unique human capacity for reason – solidified later, in Aristotle, as the possession of a rational soul – provided a capacity to recognize “the good” in its various forms and even to be able to incorporate it into one’s person or soul. In fact, there was an obligation to do so in order to have what Socrates considered a life worth living, as well as in order to be a good and contributing member of the *polis*. From Plato’s metaphor of the rational charioteer controlling the less than rational aspects of our natures to Aristotle’s brand of virtue ethics, it was clear that human beings possess significant ability to direct their actions and choices and even the responsibility to do so.

This understanding of human agency and freedom of the will, centered on the acquisition and incorporation of truth and virtue in pursuit of the improvement or even perfection of the soul, endured from classical Greece into the early Christian centuries. Even as the concept of the soul, and of its perfection, became more intensely individual over time, moral responsibility remained a hallmark of human agency. This view, however, contrasts sharply with the understanding of human agency that began to emerge in the Renaissance and which came into full flower during the Enlightenment (Taylor, 1989). This emergent view of human agency is, perhaps best captured in the quote cited above from the essayist Pico della Mirandola: “O supreme generosity of God the Father, O highest and most marvelous felicity of man! To him it is granted to have whatever he chooses, to be whatever he wills.”

In this short but heavily laden passage, the spirit of the autonomous Enlightenment agent is clearly captured. This spirit of celebration of individual autonomy continues in entirely recognizable forms into the present modern/postmodern period. First, human agency is associated with felicity, with happiness. Now, clearly, the plain sense of this association is that our having freedom of choice ought to be a source of happiness. However, once admitted, it is a very short step to the position that the function and purpose of free choice are to produce human happiness – not just in general, but in individual lives. We choose whatever we believe will make us happy. Second, agency and freedom are explicitly connected to the act of choosing. However, choosing “whatever … [we will]” is not the same thing as acquiring virtues and truth and acting accordingly (Schindler, 2017). Free will is associated with the capacity for acquisition – getting what we want, virtuous or not.^2^ And finally, the aspect of human agency that most clearly connects with the Enlightenment, and to our own time, is the notion that we can “be whatever [we] will ….” In Pico della Mirandola’s words (cited above), “with freedom of choice and with honor, as though the maker and molder of thyself, thou mayest fashion thyself in whatever shape thou shalt prefer.” Here, it is proposed that we have freedom of choice of a profound sort, the freedom and power to be makers of ourselves, and, as such, beholden only to ourselves. The nature, function, and power of the rational mind, taken to be the crowning feature of Enlightenment thinking, could hardly be expressed more clearly. The powerful rational mind, which is the source of apodictic (absolute) knowledge, is also the source of (absolute) freedom. And that freedom is manifest as power, as choice – the assertion or imposition of the individual will onto the world and even onto the self (the presumed seat and possessor of the will itself).

This tradition is the direct source of our common, modern definition of libertarian free will: Freedom of the will exists when a person in a given set of circumstances chooses response *X* but *could have* chosen another response, all circumstances remaining the same (Kane, 2005). Thus, human agency in its essential form consists in (autonomously) choosing from among alternatives. Obviously, few would seriously argue with this definition of human agency at the practical or pragmatic level – we are virtually all aware of making many perhaps hundreds of choices from among alternatives every day. Furthermore, we are all aware that, in very many cases, we really did not *have to* choose to act the way we did. We realize that we could in fact have done otherwise. However, on the theoretical, or analytical, level, this conception of human agency as free choice breaks down (see, e.g., Williams, 1992, 1994, 2005). And, it is actually quite easy to see why.

The essential defining characteristic of a choice *qua* choice is that there is a reason for it. Further, the reason somehow is sufficiently strong to “carry the day,” so to speak and determine the choice. Absent sufficient reason, no *choice* would or could emerge. Absent sufficient reason, any act would be simply a random act – produced by entirely contingent factors that just happen to be the case at the moment of “choice” (and the contemporary worldview of the social sciences offers no shortage of just such powerful abstractions as contingent causal factors). So, the traditional analysis of agency really offers only two possibilities. That is, either there is a deliberate choice based on reason, usually referred to as a free choice, or absent such reasons, the action must be understood as produced for no intelligible reason at all. At the same time, however, to the extent that reasons (and the circumstances that produce them) are strong and compelling, then to precisely that extent, one’s acts cease to be free. They must be understood as essentially compelled by the strength of whatever reasons produce them. If not, then why else would they have been chosen? We might even say, “only a fool would act otherwise.” However, in such a case, acting otherwise is not a free choice since the content of the foolishness itself prevailed and produced the choice. The conclusion here is that autonomous freedom of choice is ultimately too elusive a phenomenon to anchor and embody our innate human agency. It is also internally inconsistent and, thus, impossible in the very sense by which it is defined. In order to be a choice at all, there must be reasons for the choice that prevail over other reasons. But precisely to the extent that reasons are powerful and persuasive enough to prevail in the process leading up to the choice, the choice ceases to become genuinely free. It comes from the power of the reasons. This understanding of agency is self-contradictory – fatally so.

## An Alternative Account of Agency

There is, however, an alternative way of understanding human agency, one that is based on a conceptual foundation sturdy enough to support it, but which, ironically, requires that we essentially give up our understanding based on the hegemony of the autonomous rational self, and its ability to choose for itself and impose its will on the world, as the foundation of our agency and our human nature. The conception of the autonomous self, as it developed from Enlightenment thought, seems to work well enough at the level of praxis, in civic society, and in the law. Indeed, we all do seem to have a capacity for self-control and self-mastery in most aspects of our lives. The conception of agency as self-control or self-mastery, involving at times the deliberate imposition of will and choice, allows us to live true to a moral code, care for others, and succeed in most purposes of life. It is not, however, the foundation or fount of our human agency – which agency is the defining characteristic of humankind.

To see what this defining characteristic might be, we refer back to our earlier example of making a “free choice.” The problem with free choices being truly free is that choices are actually based on reasons, and in any decision, reasons prevail – even if, ultimately, the prevailing reason is one we might bring into the decision at the last moment just to show that we are “in charge” and can do whatever we want. *That* motive itself – to show that we are in charge – now drives the choice and, thus, renders the choice no longer a genuinely free, unfettered, autonomous one. Thus, freedom must come into the model of choice prior to any moment of deliberated choice – indeed, often long before deliberation can take place or there can be a distinct, conscious moment of choice. Agency, if it is to be real, must enter this process of living and choosing at the level of the reasons – or reason itself – not at the level of the overt choosing.^3^ Agency simply cannot be understood as free choice at the earlier level of considering reasons rather than later on at the level of choosing an action in response to those reasons because choosing among reasons, and evaluating them, is the sort of thing that itself requires reasons every bit as much as choosing an action (on down the timeline) requires reasons. So, to keep this model of free choice – we may refer to this type of deliberative conscious choice as Type 1 choice – as the *sine qua non* of our agency forces us into an infinite regress of reasons and decisions, the result of which is that we never arrive at any real point of the very autonomous freedom we take to be the *sine qua non* of human agency. Furthermore, this Type 1 choice is decidedly not the sort of choosing that characterizes the vast majority of our normal agentic lives. Very seldom in the course of daily living do we stop, enumerate, and evaluate alternative courses of actions, weigh them against each other, and then make a clear deliberative choice from among the alternatives. This is in spite of the fact that every life is composed of perhaps hundreds of agentic actions every day.

The solution to this problem really does lie at the level of our reasons for our acts, our acts of reasoning – and here we must note that by “acts” we obviously include both overt physical acts and mental acts, including evaluations, intentions, and even emotions and desires. However, the solution does not involve an act of choice of the sort that the modern/postmodern powerful rational ego – which is our legacy from Enlightenment thinking – is supposed to carry out. The Enlightenment ego imposes itself, asserts, and exercises control both upon circumstances and upon itself and chooses something or another at the expense of others. Although this choosing may happen at various times in the course of our lived experience, even several times in a single day, every time it is the same act of contemplating, deliberating, and opting from among alternatives and imposing our will on the situation. A moment’s reflection tells us, however, that for nearly all of our actions in the course of our lives, our agentive acts of choosing are not deliberative – that is, they are not the products of detached contemplation and analysis of alternatives, costs and benefits, etc. Most often, in fact, we deliberate not at all. We simply do what we intend to do, what needs to be done, what the situation calls for, in one way or another, and what makes sense. This phenomenological fact requires us to search for a type of agentive action alternative to any deliberative imposition of the will. We propose that there is such a class of actions and that these actions are more fundamental than the kind of deliberative choosing that defines the character and actions within the classical libertarian conceptions of “free” will.

It may seem paradoxical to say that the fundamental fact of human freedom is not the imposition of a choice by the will onto the world, but rather it is a sort of *yielding*, a yielding of oneself *to* a perception or conception of the world – or some aspect of our lived world. We may refer to this kind of “choosing” as Type 2 choice. The idea here is that agency entails a sort of *yielding of self* and a *taking up* of the world in a particular manner. This often produces a sense of obligation, of the sort articulated by the phenomenologist, Levinas (1969, 1990, 1995) and Williams (2002, 2005). As this yielding occurs in the lived world, it will most often take the form of *taking on* or *giving over to* something some “reading” of the world, some sense of *settling into* or *granting* of some sense or reading of our situatedness – our being-in-the-world. We might take on or give ourselves over to a person, an idea, or an ideal. We might even take on or give ourselves over to a state of affairs – empirically real or imagined – seemingly incumbent in some decision with which we are faced. Whatever the case, the agency comes not in the assertion of control (or either self or circumstance) in the making of the decision, but in what both precedes and follows from it – the actually *taking on* of some aspect of life called for by the impending decision/action or *giving ourselves over* to some implication or requirement of the decision/action. This model of agency suggests that decisions (or “free choices”) are themselves really incidences of series of ongoing *yielding to*, of *giving over* and *taking on*. To decide is to give oneself over to something, or to take something on, or take something upon us. To develop and recognize a “reason” is also to take on or give oneself over to an idea, or understanding, or purpose. This activity of giving over and taking on really has no starting point nor conclusion, and it is as continuous and as long-lived as life itself. It is, in fact life itself, lived experience. But unlike the deliberations of the autonomous, libertarian agent, it is not supposed to have a beginning or an end whereby one recognizes a choice to be made, lines up the reasons, “freely” chooses an alternative, and chooses – end of process. Rather, genuine agency (Type 2) is an infinite regress of taking on and giving over at every level of life and really at every waking moment of life. This is, quite simply, what it means to be an agent; there is neither beginning nor end. After all, if agency had some starting point, if it arose from, if it was called into being by, some non-agentic condition, physical reality, or powerful abstract ubiquitous force of one sort or another, then we would not really be agents because our agency would be derivative and qualitatively dependent (not free), and thus, it would lack meaningful substance and meaning itself.

Ultimately, what the analysis presented here means is that to be human is to be creative, and truly and always open-ended, to be the very site and source of possibility, purpose, and meaning. As Ryan and Deci (2000) note:

The fullest representations of humanity show people to be curious, vital, and self-motivated. At their best, they are agentic and inspired, striving to learn; extend themselves; master new skills; and apply their talents responsibly. That most people show considerable effort, agency, and commitment in their lives appears, in fact, to be more normative than exceptional, suggesting some very positive and persistent features of human nature (p. 68).

Furthermore, as truly agentic beings, neither our past nor our future is “fixed in place.” This is not to say that nothing about the past is fixed and given, but that the meaning dimension of the past and the meaningful tie of past to future – the “thence” and the “wherefore” – are created and maintained by agentic action itself (Williams and Gantt, 2013). In the realm occupied by meaning-making beings such as we are, we thus make and remake ourselves all the time. The lived world for us (for agentic beings of the kind we are) exists primarily as possibility and meaning. The aspect of our rationality – our legacy from Enlightenment thinking – that is most important for us is not the cold, detached, logical aspect of our human consciousness, but rather the evaluative aspect by which we can discern and judge, by many lights, the meaning and value of that which we have taken up and that to which we have given ourselves over. The moral dimension of life becomes more salient because the moral folds seamlessly into the agentic. In the positivist rational worldview, the moral requires its own set of rational and epistemological commitments, residing apart from, and in addition to, the flow of agentic living. And, thus, the moral dimensions of life are dependent upon the rational dimensions of life. In such a view, one can suspend issues of the morality of life pending definitive rational judgments about “objective” morality itself even as one’s real life, of necessity, proceeds within a moral landscape too often “fogged in” by rational uncertainty, awaiting the rational certainty that has eluded us now for centuries. This problem is at the heart of the “crisis” literature of the mid-20th century as we have described it above.

Agency, understood as the continuous taking up of the world and the giving of oneself over to the world in evaluative ways, is not to be understood as a mere attribute of the modern/postmodern Enlightenment-inspired, will imposing self. Actually, it is the other way round. Deliberative choosing and will imposing are ways of taking up the world and giving oneself over to it. Thus, the rational ego is the product of and not the source of truly agentic being-in-the-world. An astute interlocutor will no doubt observe at this point that any act of *taking up* or *giving ourselves over* can be thought of as a choice, made by a powerful rational mind as an act of free will. This is true enough. It *can* be thought of that way and agentic beings are certainly capable of acting and choosing that way. Indeed, the temptation to actually see it that way, dismissing what we have been saying about an alternative view of agency in the context of the sheer weight of the Modern rationalist tradition, might seem almost irresistible to a mind imbued with and trained in the Enlightenment thought. The problem is that agency as an act of deliberative free choice, such as an Enlightenment ego might make, is *not supposed to* end in an infinite regress – but, as we have seen, it does anyway, as we argued above. Agentive acts as conceived within the Enlightenment perspective are supposed to be self-defining and self-contained, consisting of simple stages start to finish – situation, deliberation, selection of an alternative, decision, action, and end of story. However, the implicit and necessary deliberation, selection, and decision about *reasons* for any decision to act presents another cognitive cycle of exactly the same sort as the decision to act itself (because the decision to give nontrivial credence to any reason is itself, indisputably, also an act of choice). In the end, then, every definitive answer as to how or why a particular decision to act was freely made will be question-begging, needing to invoke or presume a prior deliberation–action cycle of exactly the same sort as the original one it is supposed to explain. However, in a rationalist-conceived universe, explanations of important things such as agentic actions are not supposed to end up that way – that is, invoking a prior process as complex and almost identical to the one they are supposed to explain. In contrast, agentive acts understood as freely taking up into the self or giving oneself over to ideas and possibilities are *supposed to* entail an infinite regress because our agency is, as our life is, constant and ubiquitous, influencing and being influenced by both prior and anticipated future “taking up” and “giving over.” Life itself is a constant doing, undoing, and redoing – in the sense of being open-ended and thus agentic all the time. And, thus, one’s past and one’s future are as fluid and remediable as one’s present evaluative taking on and giving over. For human beings, it is agency all the time and all the way down. The reality of agentive action is in the very hermeneutic circularity – or spiral trajectory – of life: what’s done is done and can always be undone (or redone) for any or all of a potentially very large number of reasons which can always be taken up (or put down) and to which we might give ourselves over (or hold ourselves back).

All of this is not to say that human agency, properly understood, ends up in a chaos of random reasons and impulses that would obviate any predictive power, as has long been feared in the social sciences. On the contrary, the lifeworld in which agency lives and unfolds is not chaotic. There is no documented human drive or even proclivity toward chaos. Chaos precludes reasons and thereby destroys meaningful agency. What does become apparent upon careful analysis of human agency is that genuine understanding of the behavior of human agents will be available only from what Rychlak (1988) has described as an “introspective theoretical perspective.” This is to say that sense must be made of a person’s agentic world from the perspective of the particular agent him- or herself, rather than from an “extraspective theoretical perspective,” based on theoretical assumptions developed and applied generically and emphasizing constructs, abstractions, forces, and meat and chemical. It is not to say, however, that there can be no prediction of behavior. There may very well be consistent patterns or reasons and actions across persons, just as there are certainly patterns of consequences for behaviors. Rather, the consistency and predictability of behavior are based on the common givens and constraints and conditions of our humanity, including commonalities across persons in social and environmental realities. So, agency, as the central manifestation of our common human ontology, will also manifest itself in various commonalities of acting, living, believing, and feeling. It should be apparent in this regard that agentic living always works so much better, operating with greater breadth and depth of possibility, in the context of a reality in which there really is truth – most helpfully understood, perhaps, as the knowledge of things as they really are in their unfolding openness and meaningful possibilities (see, e.g., Heidegger, 1977). It should also be apparent that human agency works not at all in the chaos that would infuse any post-truth world such as is on offer in the currently intellectual milieu (Gantt and Williams, in press). Agency requires a source of truth and would be impotent, meaningless, and purposeless, without it (Williams, 1994).

## Concluding Thoughts on Agency as Taking up and Giving Over

The first section of this paper sketched out the predicament of the contemporary Western intellectual tradition, standing as we do on that ground where one finished form of rationalism, capped off by the German idealism descended from Hegel, met another finished form of rationalism, and capped off by the positivism descended from Comte. The result has been the creation of a “disenchanted” world (Taylor, 2007), a world where explanations of ourselves and our world are offered in terms of powerful, unseen, and immanent – that is, constituted by and constitutive of the inevitable nature of things – abstractions and structures that have real causal power in human affairs but which are discernible only to an educated intelligentsia functioning as a sort of “supermind” capable of apprehending and revealing these unseen abstractions, their manifestations, and the phenomena they produce. The influence of positivism in this intellectual activity can most clearly be seen in scientism (Sorell, 1991; Stenmark, 2001; Williams and Robinson, 2015; Gantt and Williams, 2018). In a similar vein, though not widely acknowledged, the impact of various postmodern movements can most clearly be seen in any number of (socially constructed) structures that are responsible for human behavior in any number of settings. While these movements hesitate to invoke Newtonian-style efficient causes, they still often propose the existence of certain structures and abstractions that are clearly active in the human world and move us to act, think, and feel in certain ways and serve as causal explanations for human phenomena of various sorts.

We have further argued that lost in this philosophical confluence is the flesh-and-blood human moral agent. The German philosopher, Wilhelm Dilthey (1989), recognized the inadequacy of rationalist accounts of human action in terms closely related to the argument we are making here: “No real blood flows in the veins of the knowing subject constructed by Locke, Hume, and Kant, but rather the diluted extract of reason as a mere activity of thought” (p. 50). Similarly, Dilthey’s colleague William Stern trenchantly remarked:

Of all the ways of thinking, the mathematical way is the most impersonal. The application of amount and number to personal being and doing seems to signify the reduction [of the person] to an entity merely comparable [to other entities], to a mere instance of a stiff lawfulness, in short, to a thing. It is a fact that in virtually every instance where mathematical methods – measurement, experiment, statistics – have been applied to personal life and experience as well as to cultural and social manifestations of personal communities, such a depersonalization has been the consequence. What is truly personal – the wholeness and individual specialness, the inner origin and goal-striving nature of doing – has been submerged, and persons have been made over into mere pieces of the measurable and countable larger world (cited in Lamiell, 2009, pp. 189–190).

Furthermore, in addition to the de-personalization entailed by Enlightenment-inspired rationalist accounts, the models of agency rooted in these traditions simply do not work because they inevitably end in an infinite regress of causes and reasons. The positivist tradition has not really bothered to construct theories of agency because agency cannot really exist within the causal world presumed by materialist naturalism or by the constructs, forces, structures, and laws put forth in rationalist, most post-rationalist, and structuralist accounts of any or all human actions. Post-Hegel rationalist traditions have not bothered to construct theories of agency either since agency is subsumed by the inevitability of the onward sweep of objective systemic, all-inclusive reality. Unfortunately, however, if there is no place for agency in our self-understanding, then there is no space or possibility for genuine meaning, purpose, morality, or intimacy in the human world either (Martin et al., 2003; Williams, 2005, 2017; Gantt and Williams, 2014). Indeed, in a purely naturalistic world of the sort asserted in rationalist, structuralist, and positivist accounts, there is no space even for reason itself (Lewis, 2001; Plantinga, 2011).

Postmodern positions that have found their way into the human sciences have been somewhat ambivalent about human agency. On the one hand, postmodern thinkers typically want to reject naturalist explanations of our humanity and our behavior because such perspectives clearly partake of the mistakes and excesses of Enlightenment (modernist) rationalism, including positivism and scientism, and thus risk losing track of our humanity itself. However, on the other hand, most postmodern positions, at least within the social sciences, try to insert something into that space in modernist explanatory projects traditionally occupied by laws, forces, constructs, and material biological substances – or even reason itself. Postmodernist scholars of human beings must after all account for the orderliness and structured nature of the world. For a brief period of time, structuralism, as Hayek suggested, seemed like a good compromise between rationalist scientism and chaos. However, over time, structuralism has fallen out of vogue among many postmodernists, and its most common replacement has been “discourse” or “discourse communities” (McHale, 2015). With such a move, though, we must still render an account of the nature, origin, and ontological status of “discourse” itself, as well as the effects of discourse and discourse communities upon real persons – individuals and groups. Seeking to avoid the pitfalls of proposing the powerful, individual rational ego as the source of human identity and action, many if not most postmodern thinkers are left endowing language, discourse, and community with some subtle but powerful and unseen influence (analogous to a “force”) on human thought, aspiration, and action. These forces, when discourse about them needs a name, and in instances of applied social science, as perhaps in social activism, are usually referred to as some sort of “_____ism” (e.g., racism, sexism, ethnocentrism, colonialism, heterosexism, classism). These “-isms,” we contend, often end up being very much like the structures and constructs Hayek originally warned us about and the structures and forces that pull “postmoderns” closer and closer to the mainstream of contemporary modernism of the Enlightenment of the Hegelian sort.^4^ The arguments presented in this essay comprise, in essence, a prolegomenon for the restoration of genuine human agency in the experience of our humanity and in our accounts of what it means to be a human being so that the best of what is in us as the sort of beings we are can truly unfold.

## Author Contributions

RW was the primary author on this manuscript. EG was invited to make substantial contributions to the original rough draft by expanding and revising parts of the analysis, while linking key arguments up with relevant current literature. LF was brought in to provide further revisions aimed at increasing the clarity and readability of the manuscript. All authors contributed to the article and approved the submitted version.

## Conflict of Interest

The authors declare that the research was conducted in the absence of any commercial or financial relationships that could be construed as a potential conflict of interest.

## Publisher’s Note

All claims expressed in this article are solely those of the authors and do not necessarily represent those of their affiliated organizations, or those of the publisher, the editors and the reviewers. Any product that may be evaluated in this article, or claim that may be made by its manufacturer, is not guaranteed or endorsed by the publisher.

## Footnotes

^1^All this intellectual activity also both presupposes and requires acceptance of and allegiance to the principle that true understanding will always require accepting the ubiquity and reality of abstract entities and explanation in terms of these occult operative realities.

^2^Though Pico della Mirandola, as a man of the Renaissance, might certainly have expected or at least hoped that our freedom would be used for good.

^3^Note here that it will certainly be the case that agency, at least in the way we will develop it here, can be correctly described as a “sort” of choosing, but of a nature and at a level much deeper and more intimately connected to our essential being-in-the-world than any libertarian-style conception of agency as rational, deliberated choosing from among alternatives could ever be. This discovering and remaking of our understanding of freedom and of choice will be developed in the remainder of this essay.

^4^Adequately detailed support for these arguments will need to await another forum.
